# Home-based tele-supervising rehabilitation for brain infarction patients (HTRBIP): study protocol for a randomized controlled trial

**DOI:** 10.1186/s13063-015-0585-5

**Published:** 2015-02-25

**Authors:** Wei Jin, Jing Chen, Fangfang Shi, Wuqing Yang, Yu Zhang, Yuan Liu, Wenshuai Dong, Yan Jin, Wenfeng Ma, Zhongju Ma, Xinli Min, Yin Jin, Yong Gu, Chuancheng Ren

**Affiliations:** Department of Neurology, Shanghai Number 5 Hospital, Fudan University, 801 Heqing Road, Minhang District, Shanghai, 200240 China; Shanghai NCC Electronic Co. Ltd, 68 NanSha Road, Minhang District, Shanghai, 200245 China; Shanghai Shenteng Information Technology Co., Ltd, Jingan District, 546 Yuyuan Road, Shanghai, 200040 China; China Telecom Corporation Limited Shanghai Branch, 211 Century Avenue, Pudong District, Shanghai, 200120 China

**Keywords:** Brain infarction, Stroke, Rehabilitation, Telemedicine, Telerehabilitation

## Abstract

**Background:**

With high morbidity, mortality and disability rate, brain infarction brings a huge economic and health burden to the whole society in China. Although some previous studies suggested that telerehabilitation may facilitate rehabilitation for stroke survivors at home, the evidence is insufficient for clinical application; additionally, as yet no trial evaluates efficacy of telerehabilitation for brain infarction patients. Therefore, more high quality trials are needed to provide practice evidence for this novel rehabilitation strategy.

**Methods/Design:**

Based on recruitment criteria, this assessor blinded, paralleled randomized controlled trial will recruit 210 brain infarction patients. After being randomly allocated into two groups, participants will receive home-based tele-supervising rehabilitation or conventional rehabilitation. Outcome measurement will be conducted at the end of intervention and 90-day follow-up. Among which, Barthel index assessment will be considered as primary outcome measurement, secondary outcome measurements include NIHSS score, mRS score, 3-oz water swallow test and surface electromyography. Adverse events will also be recorded during the whole process of the trial for safety assessment.

**Discussion:**

The HTRBIP trial will evaluate efficacy and safety of home-based tele-supervising rehabilitation for brain infarction patients. It is expected to provide new evidence for telerehabilitation application.

**Trial registration:**

Registration date: 17 September 2014; Registration number: ChiCTR-TRC-14005233

## Background

In China, there are 2.5 million new stroke patients each year and stroke has become a leading cause of adult death and disability [1-3]. It is estimated that stroke care annually costs nearly 40 billion RMB (6.35 billion US dollars) in China, which brings a huge health burden to the society [2]. Among different stroke types, the incidence of brain infarction is approximately twice that of hemorrhagic stroke; therefore, brain infarction may be a key element for solving the problem caused by stroke [4].

Although recombinant tissue plasminogen activator (rt-PA) has been approved as the fundamental therapy for thrombolysis, because of its utilization restrictions, the thrombolytic rate is still low [5,6]. Based on the report of the Chinese National Stroke Registry (CNSR), the rate of administration of intravenous rt-PA rate for acute brain infarction patients in China is merely 1.6% [7]; and more than half the survivors are frustrated by physical deficiency after discharge from hospital, which limits their daily activity and poses a heavy burden on their families [8]. Numerous clinical trials have demonstrated that physical rehabilitation can ameliorate neurological functional impairment and raise quality of life [9,10]. However, because of transportation restrictions and high costs, most brain infarction survivors in China cannot receive professional supervised rehabilitation, which restricts physical function recovery and delays the best rehabilitation time [11]. To resolve the problem, telerehabilitation, a novel rehabilitation strategy, has been put forward.

Telerehabilitation, which was first reported in late 1990s, can be defined as an alternative method for delivering rehabilitation services via a certain telecommunication system [12]. This novel rehabilitation strategy facilitates brain infarction survivors receiving long-term ongoing rehabilitation with supervision, especially for those who reside in remote areas and have hardly any access to rehabilitation expertise [13,14]. After being first reported in the late 1990s, telerehabilitation aroused wide interest. Several randomized controlled trials have demonstrated that compared with conventional rehabilitation care, telerehabilitation can improve physical function under remote guidance through a tele-consultant [15-17]. It also indicates that there are no differences between telerehabilitation and usual rehabilitation in patients’ satisfaction, comprehension of treatment, user-friendliness, and the patient-therapist relationship [18,19]; moreover, it may also reduce travel time, being more convenient for patients receiving rehabilitation guidance. Therefore, it seems that telerehabilitation can successfully encourage patients to persist with rehabilitation treatment and improve their physical function recovery.

Despite its advantages, clinical application for telerehabilitation is still limited. Based on a recent Cochrane study, more randomized controlled trials (RCTs) are needed to supplement clinical evidence for telerehabilitation application [20]. And because there is no clinical trial to evaluate telerehabilitation for brain infarction patients, our group will conduct an assessor blinded, randomized, paralleled controlled trial to investigate efficacy and safety of home-based tele-supervising rehabilitation for brain infarction patients, and further increase the experience gained from this novel rehabilitation strategy application.

## Methods and design

### Study objective

The main objective of the home-based tele-supervising rehabilitation for brain infarction patients (HTRBIP) trial is to investigate the efficacy and safety of home-based tele-supervising rehabilitation for patients after brain infarction. The specific study objectives are:

1. Primary research question: compared with conventional rehabilitation care, will home-based tele-supervising rehabilitation improve daily living activity of brain infarction patients at the end of 90 or 180 days after intervention has been initiated?
2. Secondary research question: compared with conventional rehabilitation care, will home-based tele-supervising rehabilitation improve neurological function of brain infarction patients at the end of 90 or 180 days after intervention has been initiated?
3. Record physiological data and adverse events to investigate safety of home-based tele-supervising rehabilitation for brain infarction patients.

### Study design

**Figure 1:**
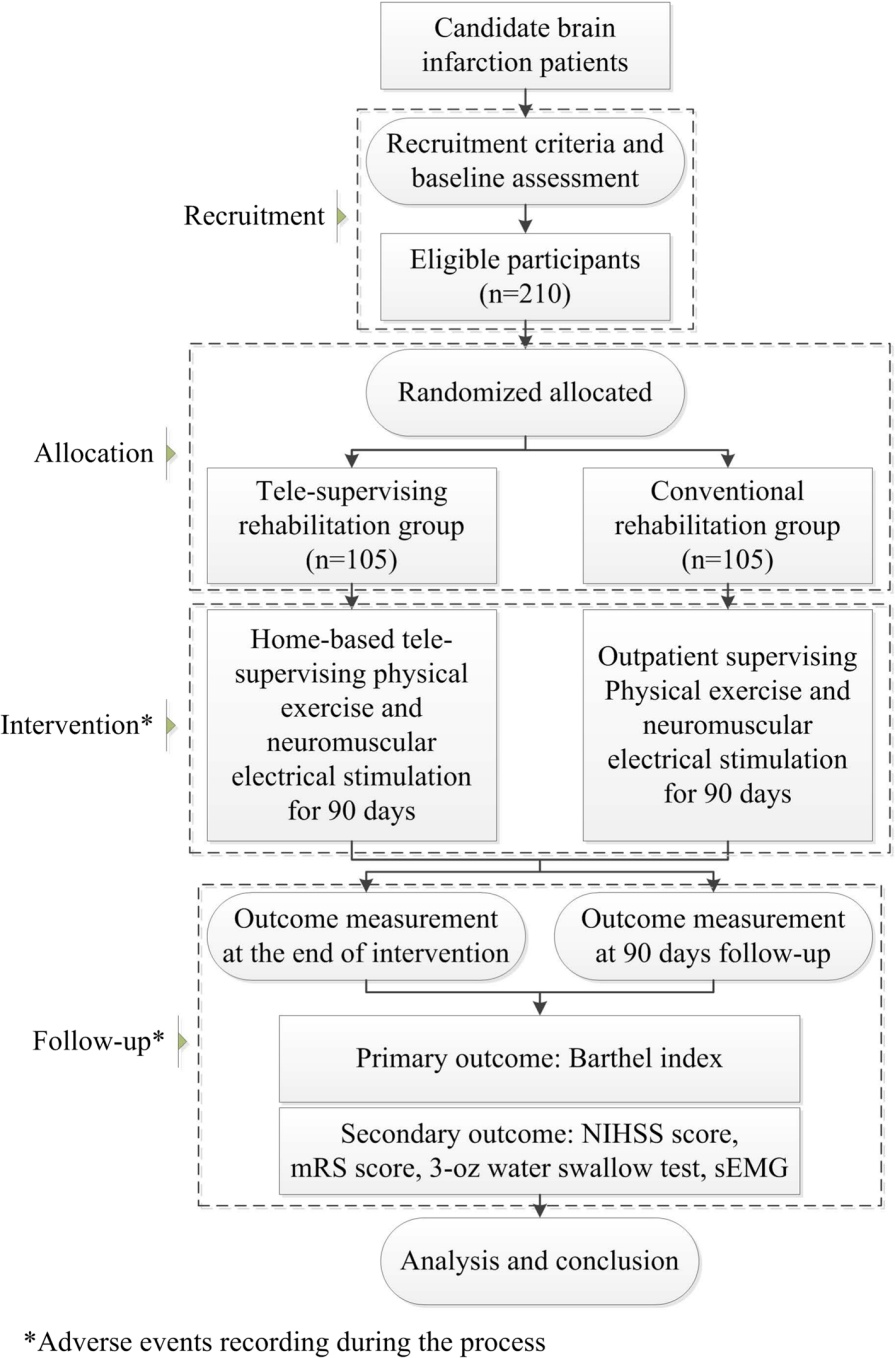
**Flow chart illustrating the home-based tele-supervising rehabilitation for brain infarction patients (HTRBIP) trial design.**

The HTRBIP trial is an assessor blinded, paralleled RCT. Participants will be recruited in Shanghai Number 5 Hospital, Fudan University, Shanghai, China. After signing informed consents, eligible patients will receive baseline assessment, and then be randomized into the tele-supervising rehabilitation group or conventional rehabilitation group. Participants in both groups will receive rehabilitation intervention for 90 days; specific rehabilitation strategy in each group will correspond to the allocation. At the end of intervention and 90-day follow-up, a group of assessors blinded to allocation and intervention will measure the outcome. Meanwhile, adverse events will be recorded during the whole process of the trial. Figure 1 depicts simply the study design, and Table 1 shows the trial procedure.

**Table 1:** **Process chart of home-based tele-supervising rehabilitation for brain infarction patients (HTRBIP) trial**

		**Time 1** ^**a**^ **: enrollment**	**Time 2** ^**b**^ **: baseline assessment**	**Time 3: intervention**	**Time 4** ^**c**^ **: at the end of intervention**	**Time 5** ^**d**^ **: 90- day follow-up**
**Informed consent obtained**		√				
**Epidemic data collection**		√				
**Medical history collection**		√				
**Physical examination**		√				
**Recruitment criteria assessment**		√				
**Randomized allocation**			√			
**Physiological data collection**	Body temperature		√	√		
	Respiratory rate		√	√		
	Pulse rate		√	√		
	Blood pressure		√	√		
	Surface electrocardiogram		√	√		
	Peripheral blood oxygen saturation		√	√		
**Primary outcome measurement** (Barthel index)			√		√	√
**Secondary outcome measurement**	NIHSS score		√		√	√
	mRS score		√		√	√
	3-oz water swallow test		√		√	√
	sEMG		√		√	√
**Adverse events record**			√	√	√	√

^a^Time 1: 2 to 7 days before intervention initiation.^b^Time 2: 0 to 2 days before intervention initiation.^c^Time 4: 90 days after intervention initiation.^d^Time 5: 180 days after intervention initiation.

Abbreviations: mRS, modified Rankin Scale; NIHSS, National Institute of Health Stroke Scale; sEMG, surface electromyography.

### Participant recruitment

Participant recruitment will be conducted in Shanghai Number 5 Hospital, Fudan University, Shanghai. Before discharge from hospital, each brain infarction patient will be identified by two independent neurologists according to inclusion and exclusion criteria. After being confirmed, eligible patients will receive a face-to-face meeting for exchanging the information of the study. If eligible patients accept and decide to participate in the study, they will be asked to sign the informed consent and follow the subsequent study process, which includes baseline assessment, randomized allocation, intervention receiving, and follow-up visiting. Informed consent from each participant should be obtained before baseline assessments.

### Sample measurement

Primary outcome of the study will be measured by using the Barthel index. The main purpose of the study is to investigate the efficacy of tele-supervising rehabilitation compared with conventional rehabilitation, and sample size calculation will be conducted by following formula:

$$ {n}_1={n}_2=\frac{2{\left({z}_{a/2}+{z}_{\beta}\right)}^2{\sigma}^2}{{\left({\mu}_1-{\mu}_1\right)}^2} $$

According to the previous study, difference in means of 90-day post-stroke Barthel index is 7.8 and standard deviation is 18.35 [21]. Taking 5% 2-side significance level and 80% power into consideration, at least 87 participants need to be recruited in each group. For 20% attrition, our study will recruit 105 participants in each group.

### Inclusion criteria

Patients will be eligible for inclusion if they meet all the following criteria: 1) be aged 35 to 85 years old, male or female; 2) be first diagnosed as brain infarction or have historical brain infarction but without neurological function impairment; 3) be 14 to 90 days from brain infarction onset; 4) have National Institute of Health Stroke Scale (NIHSS) scores from 2 to 20, modified Rankin Scale (mRS) scores from 1 to 4; 5) have not previously received any rehabilitation intervention.

### Exclusion criteria

Patients will be excluded if they meet any of following criteria: 1) have Glasgow Coma Scale (GSC) scores under 15, have been confirmed as having dementia based on Minimum Mental State Examination (MMSE) assessment, with mental disorders and unable to cooperate with examination, treatment or follow-up; 2) have disability not induced by brain infarction or disability induced by historical brain infarction; 3) have associated severe primary disease of heart, liver, kidney or hematological system; 4) have cognitive disorder, history of psychosis, substance abuse or alcoholism; 5) have skin infections in the antebrachium or crus; 6) have associated hemorrhagic or skin ulceration tendency; 7) have metal implants in the body, including cardiac pacemaker, metal stent or steel plate; 8) be in the gestation or lactation period or have a fertility plan; 9) have associated malignant tumor or severe progressive disease in any other system; 10) have been recruited by any other clinical trial in the preceding 90 days; 11) be unable to complete the basic course of treatment, with poor treatment adherence or inability to follow-up; 12) have other conditions that render them unable to continue the trial.

### Randomization and allocation concealment

After being recruited and having undergone baseline assessment, participants will be randomly allocated into either tele-supervising rehabilitation or conventional rehabilitation. Block randomization will be stratified by age (≤65 years old, > 65 years old) and NIHSS score (2 to 10, 11 to 20) with 1:1 allocation to each group. An independent statistician will conduct randomized allocation by using a computer. Allocation concealment will be ensured, as allocation information will be protected in opaque sealed envelopes by a specified staff member who is not involved in the study. Only after participant recruitment and baseline measurements are completed, will the envelopes be opened.

### Blinding

Because tele-supervising rehabilitation needs the use of a specific device for computer-based tele-consultation, it is impossible to blind the patients and tele-supervising rehabilitation therapists to allocation and intervention. In order to reduce bias, outcome assessment and data analysis will be carried out by an independent neurologist and a statistician respectively; both of them are blinded to allocation, baseline measurements, and rehabilitation intervention.

### Intervention

Participants in both groups will receive physical exercise and neuromuscular electrical stimulation after discharging form hospital. Physical exercise will be conducted for 2 hours per day following the principle of the Bobath concept, including posture control, passive movement, overturning movement, and bridge movement. Neuromuscular electrical stimulation will be conducted by using a portable muscle electricity biofeedback instrument (MyoNet-COW; NCC Electronic Co. Ltd, Shanghai, China) for 30 minutes per day. Both physical exercise and neuromuscular electrical stimulation will be carried out by trained therapists following the standard operation procedure guidance.

### Tele-supervising rehabilitation group

**Figure 2:**
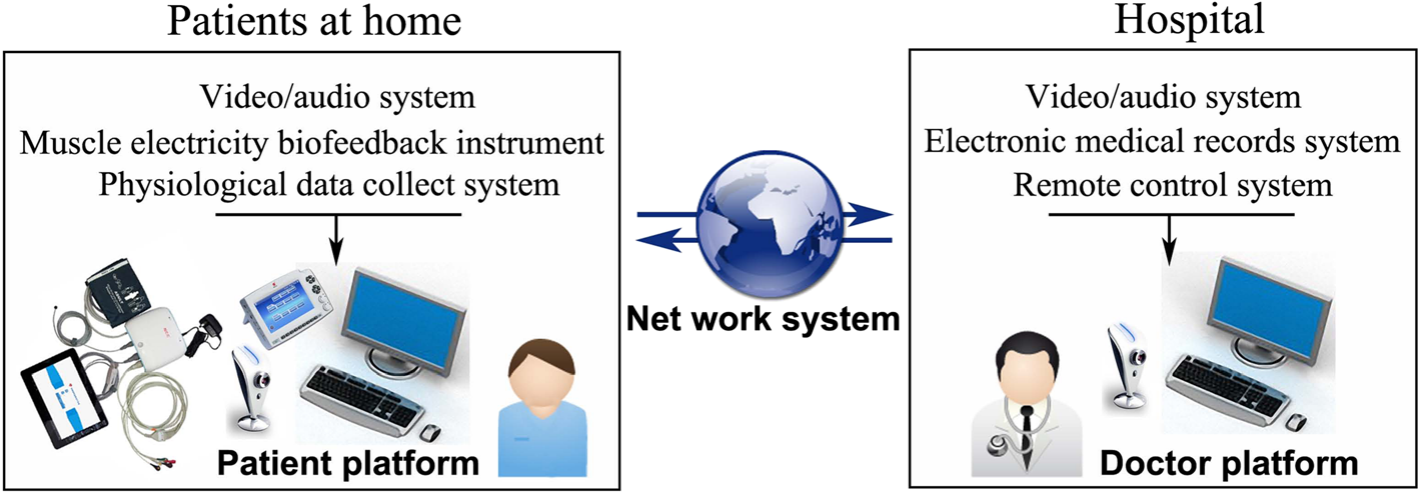
**Schematic diagram of home-based tele-supervising rehabilitation.**

Under the guidance of trained therapists and with the help of caregivers, participants in the tele-supervising rehabilitation group will receive rehabilitation therapy at their home via using a telerehabilitation system (Figure 2). The telerehabilitation system consists of three parts: a doctor work platform, a patient platform and a proprietary network system. The network system provides sufficient bandwidth for remote diagnosis, treatment and data collection. The doctor work platform is composed of a high-quality video/audio system, electronic medical records system and remote control system. Therapists can obtain the medical history information from electronic medical records, guide the participants to do the physical exercise and neuromuscular electrical stimulation by the high-quality video/audio system, and collect data by the remote control system during rehabilitation therapy. The patient platform will be implemented at participants’ homes after randomization allocation, which includes the high-quality video/audio system, physiological data collection system and muscle electricity biofeedback instrument. Participants or their caregivers will receive rehabilitation guidance from therapists via the high-quality video/audio system. Electricity biofeedback instrument will provide neuromuscular electrical simulation to the participants and collect surface electromyography (sEMG). Physiological data collection system (NTH-M6; NCC Electronic Co. Ltd, Shanghai, China) will record body temperature, respiratory rate, pulse rate, blood pressure, surface electrocardiogram, and peripheral blood oxygen saturation during the process of therapy. These physiological data will be feedback to the doctor platform for supervision by the therapists. Each participant will receive rehabilitation therapy once per day.

### Conventional rehabilitation group

Participants in the conventional rehabilitation group will receive rehabilitation therapy once per day in the outpatient department of Shanghai Number 5 Hospital, Fudan University. The therapeutic strategy is the same as the tele-supervising rehabilitation group except for using the telerehabilitation system. Physical exercise and neuromuscular electrical stimulation will be carried out face-to-face by a trained therapist at the bedside. Besides, the equipment model of the muscle electricity biofeedback instrument and physiological data collect system are the same as used in the tele-supervising rehabilitation group.

### Data measurement

Data measurement will be carried out by trained independent assessors, who are blinded to allocation and intervention, and who will be required to be prevented from learning allocation or intervention information from patients during outcome measurements. Baseline data assessment will be carried out in hospital before randomization allocation. In order to keep allocation and intervention from assessors, outcome measurements will be conducted at the neurology outpatient department at the end of intervention and 90-day follow-up. A specified trial staff member will make an appointment with patients the day before outcome measurements and will make suggestion about the next day’s physical examination including not to provide treatment information to the physician.

### Baseline assessment

Baseline assessment consists of epidemiologic data (including age, gender, history of present complaint, past medical history, physical examination, and auxiliary examination); NIHSS score, Barthel index score, mRS score, water swallow test, and sEMG. Meanwhile, physiological data will also be measured, which includes body temperature, respiratory rate, pulse rate, blood pressure, surface electrocardiogram, and peripheral blood oxygen saturation.

### Primary outcome measurement

Primary outcome measurement will be conducted by using the Barthel index at the end of intervention and 90-day follow-up. The Barthel index consists of ten items: presence or absence of fecal incontinence, presence or absence of urinary incontinence, help needed with grooming, toilet use, feeding, transfers, walking, climbing stairs, bathing and dressing. The score ranges from 0 to 100, with high scores demonstrating high activities of daily living [22,23].

### Secondary outcome measurement

Secondary outcome measurement, which includes NIHSS score, mRS score, 3-oz water swallow test and sEMG, will be carried out at the same time with the primary outcome measurement.

Neurological function deficient will be measured by NIHSS score; it has been confirmed as a widely used scale with high reliability and good convergent validity. NIHSS includes 11 items, ranging from 0 to 42, with low scores depicting good neurological function [24,25].

General disability condition will be measured by mRS score, which consists of 6 ranks: rank 0 demonstrates no disability symptoms; rank 1 demonstrates no significant disability, despite with symptoms, able to perform all usual duties and activities; rank 2 demonstrates slight disability, although with the ability to look after own affairs without assistance, but be unable to carry out all previous activities; rank 3 means moderate disability, requiring some help in daily life, but with the ability to walk without assistance; rank 4 means moderately severe disability, with the inability to walk without assistance and attend to own bodily needs without assistance; rank 5 means severe disability, depicts patients who are bedridden, incontinent or require constant nursing care; rank 6 equates to death [26,27].

Swallowing function and the presence of dysphagia will be measured by the 3-oz water swallow test [28]. Participants will be asked to drink 3 oz of warm water without interruption. Criteria for assessment of swallowing includes being unable to complete the task, coughing, choking, or with a wet-hoarse vocal quality exhibited either during or within 1 minute of test completion. Compared with other bedside assessment, the 3-oz water swallow test has high specificity and sensitivity in detecting aspiration in dysphagia patients after stroke [29].

As motor unit recruitment is crucial for evaluating muscle activity after brain infarction, sEMG will be employed to monitor the root mean-squared (RMS) value of target body regions. RMS value of the sEMG signals reflects the association of muscle activity with motor unit recruitment [30].

### Adverse events

Although therapeutic treatment will be conducted with the guidance of trained therapists, and the intervention strategy of the study is low risk, it is impossible to ignore adverse event measurement. Therefore, all the participants as well as their caregivers and research team staff will be asked to report adverse events. Besides, physiological data will be recorded to assess the physical condition of the participants during intervention. A group of research staff will be trained to record and analyze physiological data and deal with adverse events. If severe adverse events happen, the group will report the events to the study director and quality control committee and the study will be suspended if necessary. Once the study has to be suspended because of severe adverse events, only when the severe adverse events can be successfully prevented, can the suspended study be recommenced.

### Quality control

The study has been registered in the Chinese Clinical Trial Registry (ChiCTR) and supervised by the superior authority. A Quality Control Committee will be established before trial initiation, and will consist of six members, including two neurologists, two rehabilitation specialties, and two statisticians. The committee will help the research team write the trial protocol and standard operation procedure, supervise the whole process of the study, and control the quality. The research team will receive specific training for standardizing the procedure of the entire study including participant recruitment, therapeutic intervention, outcome measurement and data analysis. Additionally, all the data should be checked according to the standard operation procedure of the study at least once per week during the study process.

### Statistics

An independent statistician, who is blind to allocation and intervention, will take charge of data analysis. All the variables will be analyzed according to an intent-to-treat (ITT) basis, and the statistical tests will be 2-side and at the 5% significance level. All the participants under randomization will be included in the analysis, regardless of whether or not they adhered to the treatment or provided complete data sets. For providing valid inferences with less restrictive assumptions, multiple imputation will be performed to analyze missing data. Continuous variables will be summarized by means and standard deviation. The normality of the distribution will be checked before comparison by using the Shapiro-Wilk test. Normally distributed variables will be compared by using the *t*-test, and the Mann-Whitney *U-*test will be performed to compare non-normally distributed variables. Discrete variables will be summarized by relative frequencies and analyzed by using a non-parametric test. The level of statistical significance will be set at *P* < 0.05. SAS V9.1 (SAS Institute, Cary, NC, USA) will be employed for all the statistical analysis. The statistical analysis plan will be fully specified and approved before unmasking.

### Ethics

The HTRBIP trial follows the Helsinki Declaration and has been approved by the Independence Ethics Committee of the Fifth Peoples’ Hospital of Shanghai, Fudan University (ethic approval number: 2014-ETRE-066). Participants will be acquainted with informed consent and asked to sign for it before being recruited.

### Dissemination policy

The HTRBIP trial study and results will be published in peer-reviewed journals in line with the reporting guidelines; namely, TIDieR for description of interventions and the Consolidated Standards of Reporting Trials (CONSORT) extension for non-pharmacological drugs. Trial results will be submitted to the trial registry, and will be communicated to participants via written and oral reports. The results will also be reported to local policy-makers (Shanghai Municipal Commission of Economy and Informatization, Shanghai Municipal Commission of Health and Family Planning). Additional stakeholders, including telerehabilitation device manufacturers and network service corporations, will be informed of the trial findings. Trial results will be disseminated regardless of the magnitude or direction of effect.

## Discussion

Stroke is the leading cause of adult death and disability in China, and brain infarction is its majority component. Since over 50% of brain infarction patients are still suffering from physical disabilities after discharge from hospital [20], and because most brain infarction survivors in China used to conduct rehabilitation at home, a novel rehabilitation strategy is needed to facilitate rehabilitation in patients’ residences under professional supervision.

Home-based telerehabilitation, which utilizes information and communication technology to connect patients and therapists, is considered as a potential solution for poor rehabilitation outcome of brain infarction patients after discharging from hospital [31,32]. Some research groups conduct clinical trials to investigate the feasibility of home-based physical rehabilitation. To date, there are ten RCTs investigating post-stroke telerehabilitation [20]. However, because of small sample size, significant heterogeneity and high risk bias; these previous RCTs could not conclude the practical guidance for post-stroke telerehabilitation application; thus, more adequately powered studies are required. Additionally, because there are currently no clinical trials evaluating the effectiveness of telerehabilitation for brain infarction patients worldwide, it is reasonable for us to conduct this clinical trial to provide new evidence for home-based tele-supervising rehabilitation application.

The HTRBIP trial is designed as an assessor blinded, paralleled RCT. Tele-supervising rehabilitation will be conducted through a specialized system that can connect therapist and patient over distance; data collecting will be simultaneously performed during rehabilitation. Barthel index at the end of intervention and at 90-day follow-up will be evaluated as primary outcome measurement, which represents the general physical function for disabled patients after brain infarction. NIHSS score, mRS score, 3-oz water swallow test and sEMG will be employed for secondary outcome measurement. Furthermore, adverse events will be recorded for safety evaluation. For tele-supervising rehabilitation specialized remote interaction devices are required, and because it is impossible to blind the intervention strategy to the patients and therapists, this trial could not be double-blinded. To make up for this limitation and ensure the quality of the trial, outcome assessors and data statisticians will be blind to the allocation and intervention strategy.

The HTRBIP trial will evaluate efficacy and safety of home-based tele-supervising rehabilitation for brain infarction survivors in China, which is expected to supplement evidence for telerehabilitation application, and increase the experience gained for its practice.

## Trial status

Recruitment has begun as of September 2014.

## Abbreviations

ChiCTR: Chinese Clinical Trial Registry, CONSORT, Consolidated Standards of Reporting Trials
CNSR: Chinese National Stroke Registry
GSC: Glasgow Coma Scale
HTRBIP: Home-based tele-supervising rehabilitation for brain infarction patients
ITT: Intention-to-treat
MMSE: Minimum Mental State Examination
mRS: Modified Rankin Scale
NIHSS: National Institute of Health Stroke Scale
RCT: Randomized controlled trial
RMS: Root mean-squared
rt-PA: Recombinant tissue plasminogen activator
sEMG: Surface electromyography
